# Supplementary material for: The prognostic value of preoperative neoindices consisting of lymphocytes, neutrophils and albumin (LANR) in operable breast cancer: a retrospective study
Source: PeerJ. 2024 May 15;12:e17382. doi: 10.7717/peerj.17382 (PMC11102052; doi:10.7717/peerj.17382)
Supplement: Supplemental Information 1 [file peerj-12-17382-s001.doc]

STROBE Statement—checklist of items that should be included in reports of observational studies

|  | Item  No | Recommendation |
| --- | --- | --- |
| **Title and abstract** | 1 | **The Prognostic Value of Preoperative Neoindices Consisting of Lymphocytes, Neutrophils and Albumin (LANR) in Operable Breast Cancer：A Retrospective Study** |
|  |  | (1) We analyzed the relationship between the LANR and prognosis of patients with breast cancer.  (2) A high preoperative LANR may be an independent prognostic factor for breast cancer. |
| Introduction | | |
| Background/rationale | 43 | Preoperative inflammatory factors and nutritional status are strongly associated with the prognosis of a variety of cancers. We explored the relationship between preoperative lymphocytes, neutrophils and albumin (LANR) and progression-free survival in breast cancer patients. |
| Objectives | 25 | We explored the relationship between preoperative lymphocytes, neutrophils and albumin (LANR) and progression-free survival in breast cancer patients. |
| Methods | | |
| Study design | 76 | a retrospective study |
| Setting | 76 | We retrospectively collected clinical and pathologic information on 200 female breast cancer patients who underwent their first surgical treatment for breast cancer from January 2015 to December 2019 at the Affiliated Hospital of Jiangnan University.  The inclusion criteria were as follows: (1) all patients had a pathologic diagnosis of primary breast cancer; (2) patients with histologically confirmed non-metastatic invasive female breast cancer; (3) all underwent radical surgical resection for the first time; (4) had complete clinicopathologic and laboratory data.  Exclusion criteria: (1) patients with a history of malignancy or a combination of other primary tumors; (2) patients with acute and chronic inflammation and infection prior to surgery; (3) patients who had received neoadjuvant therapy such as radiotherapy prior to surgery; |
| Participants | 76 | We retrospectively collected clinical and pathologic information on 200 female breast cancer patients who underwent their first surgical treatment for breast cancer from January 2015 to December 2019 at the Affiliated Hospital of Jiangnan University. |
|  |  |  |
| Variables | 41 | LANR, lymphocytes × albumin/neutrophils |
| Data sources/ measurement | 94 | Body mass index (BMI) was classified as <18.5, 18.5-23.9, and >23.9. Cancer stage (including tumor size, positive axillary lymph nodes, and TNM) was assessed for each patient according to the American Joint Committee on Cancer (AJCC) staging manual, eighth edition. Serum biochemical tests, including laboratory data (lymphocytes, neutrophils and albumin), were performed at the preoperative baseline follow-up. |
| Bias |  | None |
| Study size | 76 | We retrospectively collected clinical and pathologic information on 200 female breast cancer patients who underwent their first surgical treatment for breast cancer from January 2015 to December 2019 at the Affiliated Hospital of Jiangnan University. |
| Quantitative variables | 103 | Independent samples t-test or Wilcoxon test was used to compare differences between groups of continuous variables. The chi-square test or Fisher's exact test was used to calculate differences between categorical variables. |
| Statistical methods | 103 | SPSS 26.0 software was used for statistical analysis. Independent samples t-test or Wilcoxon test was used to compare differences between groups of continuous variables. The chi-square test or Fisher's exact test was used to calculate differences between categorical variables. Subject work characteristic (ROC) curves were used to convert continuous variables (lymphocytes, neutrophils, albumin, and LANR) to dichotomous variables using the inflection point as the threshold. In survival analysis, Kaplan-Meier survival curves and log-rank tests were used to compare survival differences between groups classified by dichotomous biochemical indicators. Cox proportional risk regression models were used for univariate and multivariate regression analyses. Subgroup analyses were performed to show the prognostic correlation between patients with different characteristics and the new index. All analyses were two-sided, and *p* values <0.05 were considered statistically significant. |
|  |  |  |

| Results | | |
| --- | --- | --- |
| Participants | 114 | A total of 200 breast cancer patients, all of whom eventually underwent surgery, were included in this study, and the demographic and clinicopathological characteristics are shown in Table 1. The median age of all patients was 55.5 years (range, 50-62 years). According to the AJCC classification, there were 82 patients (41%) with stage I, 70 patients (35%) with stage II, and 48 patients (24%) with stage III. 7 (3.5%) were underweight, 94 (47%) were normal weight, and 99 (49.5%) were overweight. In terms of tumor size, 101 patients (50.5%) had T1, 96 patients (48%) had T2, and 3 patients (1.5%) had T3. 126 patients (63%) had negative axillary lymph nodes, 38 patients (19%) had 1 to 3 positives, 21 patients (10.5%) had 4 to 9 positives, and 15 patients (7.5%) had more than 10 positives. 130 patients (65%) had positive ER status，The ER status was positive in 65% of patients and negative in 70 (35%). 98 patients (94%) had positive PR status and 102 (51%) were negative. For Her-2 status, 163 (81.5%) showed positive and 37 (18.5%) negative. A total of 69 patients (34.5%) relapsed and 131 patients (65.5%) did not relapse. The median follow-up time was 46 months. |
| Descriptive data | 49 | (a) Age, BMI, Tumor size, TNM stage, Node positivity, ER, PR, HER-2, lymphocyte, neutrophils, albumin |
| Outcome data |  | This is a Retrospective Study, this item is not applicable. |
| Main results | 94 | Body mass index (BMI) was classified as <18.5, 18.5-23.9, and >23.9.  Cancer stage (including tumor size, positive axillary lymph nodes, and TNM) was assessed for each patient according to the American Joint Committee on Cancer (AJCC) staging manual, eighth edition. |
|  |  | Tumor size (＜2cm、＞2cm,＜5cm、＞5cm). Node positivity (0、1~3、4~9、＞10) ER、PR、HER-2（Positive、Negative） |
| Other analyses | 110 | Cox proportional risk regression models were used for univariate and multivariate regression analyses. Subgroup analyses were performed to show the prognostic correlation between patients with different characteristics and the new index. |
| Discussion | | |
| Key results | 202 | LANR is an essential predictor for breast cancer patients and provides a therapeutic basis for clinicians and patients. |
| Limitations | 192 | This study has several limitations. First, it was a single-center study with a small sample size (200 cases), and the findings may not be sufficient to fully reflect the status of breast cancer. Our research team aimed to perform a multicenter data analysis on a large prospective study sample to further validate the role of LANR in assessing breast cancer prognosis. Second, this is a retrospective study with certain inherent limitations. Third, the survival endpoint was defined as progression-free survival of patients with a median follow-up time of 46 months, which is too short a follow-up time and lacks OS, and the results may differ from those studies using tumor-related death as a survival endpoint. Fourth, we evaluated LANR at only one time point before surgery. LANR changes throughout the patient's cancer treatment cycle may have a more significant impact on prognosis, but we did not evaluate it in our study. |
| Interpretation |  |  |
| Generalisability | 203 | In summarization, the data from this study indicates that LANR will serve as a new indicator of good prognostic performance affecting patients with breast cancer. LANR may therefore be a useful biomarker in breast cancer assessment to guide appropriate treatment, adjust follow-up intervals and improve prognosis. |
| Other information | | |
| Funding | 208 | This work was supported by the Wuxi Taihu Lake Talent Plan, Supports for Leading Talents in Medical and Health Profession（Mading academician, 4532001THMD), Beijing Bethune charitable Foundation(2022-YJ-085-J-Z-ZZ-011), Top Talent Support Program for Young and Middle-aged people of Wuxi Health Committee (BJ2020047). |

*Give information separately for cases and controls in case-control studies and, if applicable, for exposed and unexposed groups in cohort and cross-sectional studies.

**Note:** An Explanation and Elaboration article discusses each checklist item and gives methodological background and published examples of transparent reporting. The STROBE checklist is best used in conjunction with this article (freely available on the Web sites of PLoS Medicine at http://www.plosmedicine.org/, Annals of Internal Medicine at http://www.annals.org/, and Epidemiology at http://www.epidem.com/). Information on the STROBE Initiative is available at www.strobe-statement.org.
